# Chemical carcinogens: implications for cancer treatment

**DOI:** 10.1172/JCI174319

**Published:** 2023-10-16

**Authors:** Shaofeng Liu, Mary Saunders, Tak W. Mak

**Affiliations:** 1 Princess Margaret Cancer Centre, University Health Network, Toronto, Ontario, Canada.; 2 Departments of Medical Biophysics and Immunology, University of Toronto, Toronto, Ontario, Canada.; 3 Department of Pathology, School of Clinical Medicine, Li Ka Shing Faculty of Medicine, The University of Hong Kong, Hong Kong, China.; 4 Centre for Oncology and Immunology, Hong Kong Science Park, Hong Kong, China.

## Abstract

Carcinogen exposure has been associated with enhanced cancer immunogenicity that is often attributed to neoantigen generation. However, the broader, neoantigen-independent impact of carcinogens on immune responses to cancer cells remains underexplored. In this issue of the *JCI,* Huang et al. uncover a mechanism wherein carcinogen-treated cancer cells exhibit an inability to establish an immunosuppressive tumor microenvironment (TME) due to reduced M-CSF expression. Intriguingly, the so-called carcinogen-induced tumor-associated macrophages (TAMs) within this TME exhibited anti-tumor properties instead of the conventional immunosuppressive phenotype. This phenomenon extended to human lung cancers, as evidenced by TAM reprogramming in smokers versus nonsmokers. This study substantially advances our understanding of carcinogen-mediated effects on cancer immunogenicity, potentially redirecting approaches to cancer immunotherapy.

## Carcinogen treatment augments cancer immunogenicity beyond neoantigens

Carcinogens are substances with the ability to cause cancer mainly by generating mutations in cellular DNA. Carcinogens responsible for a substantial number of cancer types include high-energy radiation (e.g., UV light and X-ray irradiation), various chemicals (e.g., components of tobacco smoke, alcohol, and formaldehyde), and oncogenic viruses (e.g., HPV and HBV) (1, 2). Lung cancer is the prime example of a carcinogen-caused tumor, since 90% of these malignancies are attributable to tobacco exposure (3). Components of tobacco smoke induce driver mutations in human bronchial epithelial cells, resulting in an increased mutational burden and rampant cell-to-cell heterogeneity (3). Because carcinogen-induced malignancies contain relatively more neoantigens due to their higher mutational burden, these cancer types intrinsically exhibit enhanced immunogenicity compared with spontaneous tumors (4). However, the DNA damage and genomic instability induced by carcinogens have additional numerous effects and can lead to innate immune activation and/or alterations to the epigenetic landscape of the genome (5–7). Delineation of the effects of these neoantigen-independent mechanisms of carcinogens on cancer immunogenicity is in its infancy.

In this issue of the *JCI*, Huang and colleagues present an investigation of the impact of a carcinogen on cancer cell immunogenicity (8). They first treated the MMTV-PyMt^tg^ (PyMt) breast cancer cell line with the carcinogen 7,12-Dimethylbenz[a]anthracene (DMBA), and selected clones (PyMt-DMBA3-4; hereafter denoted as “DMBA3-4 cells”) that had a growth rate comparable to that of control cells treated with DMSO (PyMt-DMSO3-1; hereafter denoted as “DMSO3-1 cells”.) DMBA3-4 cells showed greater immunogenicity, as they were rejected by T cells in immunocompetent mice, as expected. However, when the authors sequenced the genome of these DMBA3-4 cells, they found no missense single nucleotide variants (SNVs), suggesting that their high level of immunogenicity could not be attributed to an increased neoantigen load derived from missense mutations. To preclude any influence of potential DMBA-induced neoantigens, the authors injected WT mice that had rejected DMBA3-4 cells (i.e., immune mice) with DMSO3-1 cells lacking any neoantigens. The immune mice rejected the DMSO3-1 cells, indicating that neoantigen-specific T cells were not required for the rejection phenotype. The authors then confirmed this finding with an even more convincing coinjection experiment. In this setting, the authors injected naive WT mice with DMBA3-4 and DMSO3-1 cells contralaterally, and found that the DMSO3-1 cells were not only not rejected but even blocked rejection of the DMBA3-4 cells. The authors then injected a mixture of DMBA3-4 and DMSO3-1 cells into one site on WT mice and observed that the DMSO3-1 cells completely prevented the rejection of DMBA3-4 cells. These results elegantly demonstrated that DMSO3-1 cells locally and systemically prevented the rejection of DMBA3-4 cells, implying that the immunogenicity of DMBA3-4 cells is likely driven by the loss of their ability to establish an immunosuppressive TME rather than by the gain of an immunogenic factor (8).

## Carcinogen-treated cancer cells reprogram tumor-associated macrophages

To identify the cell types that were responsible for the poorly immunosuppressive TME in DMBA3-4 tumors, Huang and colleagues analyzed tumor-derived CD45^+^ leukocytes by flow cytometry. They detected a decrease in F4/80^+^ and CD11b^+^F4/80^+^ TAMs in DMBA3-4 tumors compared with DMSO3-1 tumors. Indeed, depletion of these macrophages in DMSO3-1 tumors suppressed cancer cell growth in WT mice, confirming their immunosuppressive nature in the DMSO3-1 TME. When the authors examined the transcriptional profiles of these TAMs, they observed increases in proinflammatory genes such as *Cxcl9, Cxcl10, Cxcl11, Prf1,* and *Gzmb* in DMBA3-4 TAMs compared with DMSO3-1 TAMs. These data suggested that carcinogen-treated tumors could reprogram the TME (8).

What caused the reduction and reprogramming of TAMs in DMBA3-4 tumors? To answer this question, the authors examined the secretome profiles of DMBA3-4 and DMSO3-1 cells. Culture supernatants of DMBA3-4 cells showed elevated levels of CCL5, CCL17, CXCL10, and osteoprotegerin, but reduced levels of M-CSF and osteopontin (OPN). The authors then determined that a decrease in M-CSF was responsible for the reprogrammed TME in DMBA3-4 tumors. Indeed, macrophage migration toward DMBA3-4 tumor cells was notably lower than that toward DMSO3-1 cells*,* and blockade of the M-CSF receptor abrogated any differences in macrophage migration toward DMBA3-4 versus DMSO3-1 cells in an in vitro migration assay (8).

In addition to evaluating secreted proteins, Huang and colleagues examined the expression of various inhibitory ligands (PD-L1, PD-L2, CD155, and CD112) on the surface of DMBA3-4 cells. They found a dramatic reduction in surface CD155 on DMBA3-4 cells compared with DMSO3-1 controls. To determine the functions of M-CSF and CD155 in mediating TME reprogramming in DMBA3-4 tumors, the authors used antibodies to block the two pathways mediated by M-CSF- or CD155-mediated signaling. Treatment with a combination of anti-CSFR1 and anti-TIGIT antibodies led to the complete rejection of 8 of 10 DMBA3-4 plus DMSO3-1 tumors in WT mice, demonstrating that M-CSF and CD155 play redundant roles in driving the profound reversal of immunosuppression in the TME of a carcinogen-treated tumor (8).

## Implications for human cancers

**Figure 1:**
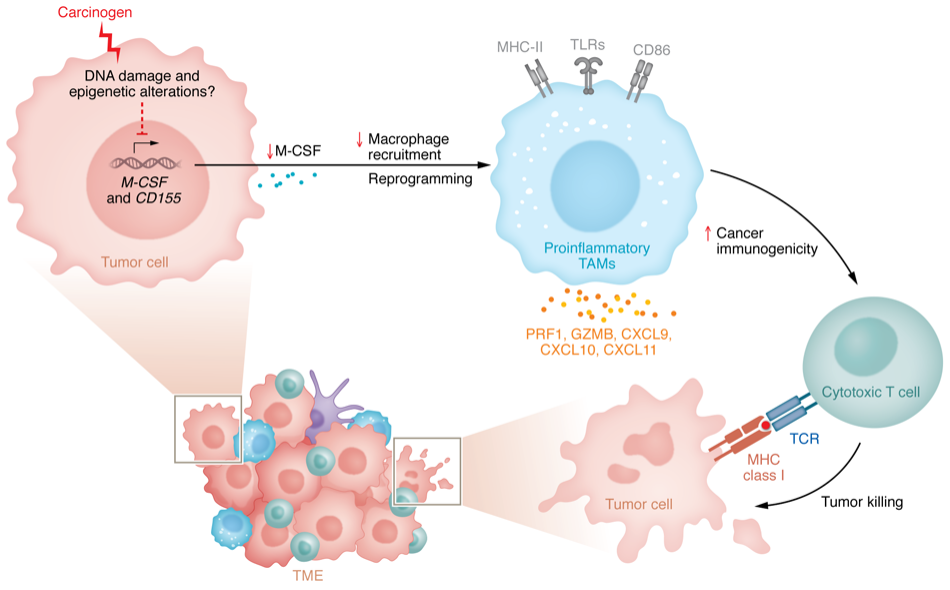
Carcinogens reprogram the TME by downregulating cancer cell expression of M-CSF and CD155. Carcinogen exposure was thought to enhance cancer cell immunogenicity mainly by generating a high mutation burden and promoting neoantigen expression. Huang and colleagues found a mechanism separate from neoantigen production. Carcinogen-treated cancer cells reduced their expression of M-CSF and CD155 and increased immunogenicity by reprogramming TAMs within the TME. Carcinogen exposure resulted in the reduced recruitment of TAMs. In addition, TAMs were reprogrammed to express the M1 markers MHC-II, TLRs, and CD86, and proinflammatory mediators including PRF1, GZMB, CXCL9, CXCL10, and CXCL11. These proinflammatory TAMs increased tumor immunogenicity via T cell–mediated cytotoxicity, resulting in cancer cell killing (8).

To determine whether carcinogen-induced TME reprogramming also occurs in human cancers, the authors analyzed single-cell RNA-Seq (scRNA-Seq) data obtained from 34 lung cancers in an annotated cohort of former smokers and individuals who had never smoked. TAMs in former smokers showed a carcinogen-induced phenotype, whereas TAMs in individuals who had never smoked displayed a more classical suppressive TAM signature. In addition, expression levels of *CSF1* and *PVR* were lower in former smokers compared with those who had never smoked. These results suggest that carcinogen-exposed human lung cancer cells can also reprogram TAMs, which may contribute to the increased lung cancer immunogenicity observed in smokers. These findings provide compelling evidence that carcinogen exposure leads to reduced immunosuppression due to the reprogramming of TAMs, an event independent of neoantigen generation (8) (Figure 1). Although it is hard to imagine that one might seek to increase the sensitivity of a patient’s tumor to immunotherapy by applying a toxic carcinogen, the work of Huang et al. may still suggest that targeting this TME-reprogramming mechanism may provide an alternative approach to treat at least some cancers.

## Unanswered questions

Huang et al. have made unexpected findings based on solid experimentation, and yet many questions remain unanswered. For example, how does carcinogen treatment decrease M-CSF and CD155 expression? The primary effect of a carcinogen is the induction of DNA damage. Whether the M-CSF and CD155 gene loci suffered DMBA-induced DNA damage that altered their expression, or whether these reduced expression levels were secondary to changes caused by disruption of chromatin regulators needs to be further explored. Another question is: do carcinogens have DNA damage–independent effects on cancer cell gene expression, such as epigenetic reprogramming of tumor cells? In addition, do these changes to the TME occur in response to all carcinogens and affect all cancer types? The answer to this last question is “probably not”, given the highly heterogeneous nature of malignancies, meaning that defining the carcinogens that can reprogram each tumor type will require individual investigation. The final but possibly most important question is: how does one translate the discoveries made by Huang et al. into the clinic? Many chemotherapy drugs resemble carcinogens in that they also can induce DNA damage; indeed, there is a substantial overlap between anticancer drugs and carcinogens (9). The combinations of chemotherapy plus immunotherapy that are now frequently used in clinical trials for treating various cancer types are based on the rationale that the chemotherapy component may induce the generation of more targets (e.g., neoantigens) for attack by the immunotherapy component (10, 11). Thus, it is a matter of great urgency to determine the neoantigen-independent effects of chemotherapy on the TME and the nature of the mechanisms driving these effects. With this knowledge, better combination regimens of chemotherapy plus immunotherapy can be designed to benefit cancer patients.

## Version 1 10/16/2023

Electronic publication
